# Brain Magnetic Resonance Imaging Phenome-Wide Association Study With Metal Transporter Gene *SLC39A8*

**DOI:** 10.3389/fgene.2021.647946

**Published:** 2021-03-15

**Authors:** Evan R. Hermann, Emily Chambers, Danielle N. Davis, McKale R. Montgomery, Dingbo Lin, Winyoo Chowanadisai

**Affiliations:** Department of Nutritional Sciences, Oklahoma State University, Stillwater, OK, United States

**Keywords:** ZIP8, PheWAS, GWAS, manganese, zinc, iron, nutrigenomics, nutrition

## Abstract

The *SLC39A8* gene encodes a divalent metal transporter, ZIP8. *SLC39A8* is associated with pleiotropic effects across multiple tissues, including the brain. We determine the different brain magnetic resonance imaging (MRI) phenotypes associated with *SLC39A8.* We used a phenome-wide association study approach followed by joint and conditional association analysis. Using the summary statistics datasets from a brain MRI genome-wide association study on adult United Kingdom (UK) Biobank participants, we systematically selected all brain MRI phenotypes associated with single-nucleotide polymorphisms (SNPs) within 500 kb of the *SLC39A8* genetic locus. For all significant brain MRI phenotypes, we used GCTA-COJO to determine the number of independent association signals and identify index SNPs for each brain MRI phenotype. Linkage equilibrium for brain phenotypes with multiple independent signals was confirmed by LDpair. We identified 24 brain MRI phenotypes that vary due to MRI type and brain region and contain a SNP associated with the *SLC39A8* locus. Missense ZIP8 polymorphism rs13107325 was associated with 22 brain MRI phenotypes. Rare ZIP8 variants present in a published UK Biobank dataset are associated with 6 brain MRI phenotypes also linked to rs13107325. Among the 24 datasets, an additional 4 association signals were identified by GCTA-COJO and confirmed to be in linkage equilibrium with rs13107325 using LDpair. These additional association signals represent new probable causative SNPs in addition to rs13107325. This study provides leads into how genetic variation in *SLC39A8*, a trace mineral transport gene, is linked to brain structure differences and may affect brain development and nervous system function.

## Introduction

Manganese is an essential mineral for neurodevelopment and brain function, although high manganese exposure can lead to neurotoxicity that can manifest as parkinsonism and psychosis (Horning et al., 2015). The SLC39 (ZIP) gene family consists mostly of zinc transporters but some ZIP transporters are permeable to other minerals such as iron and manganese (Eide, 2004). Inactivating mutations in the metal transporter gene *SLC39A8*, which codes for the transporter protein ZIP8, result in intellectual disability, cranial malformations, cerebellar atrophy, hypotonia, low blood manganese, and impaired glycosylation (Boycott et al., 2015; Park et al., 2015). These mutations impair manganese uptake and result in reduced manganese in mitochondria and increased oxidative stress (Choi et al., 2018). ZIP8 has the ability to transport multiple metals, including zinc (Wang et al., 2012), iron (Wang et al., 2012), and cadmium (Dalton et al., 2005), in addition to manganese (He et al., 2006).

One non-synonymous polymorphism rs13107325 results in a substitution of threonine for alanine at amino acid 391 and is most prevalent in populations of European ancestry (Carrera et al., 2012). The minor allele of single-nucleotide polymorphism (SNP) rs13107325 (T, threonine) has reduced manganese transport activity in HEK-293T cells (Haller et al., 2018). Subjects homozygous for rs13107325 have low whole blood manganese (Ng et al., 2015) and plasma manganese (Haller et al., 2018) concentrations and altered protein glycosylation (Lin et al., 2017). This missense ZIP8 polymorphism is associated with pleiotropic effects including childhood neurodevelopment and behavioral problems (Wahlberg et al., 2018), lower fluid intelligence (Hill et al., 2019), and greater risks for schizophrenia (Carrera et al., 2012; Schizophrenia Working Group of the Psychiatric Genomics Consortium, 2014; Pickrell et al., 2016) and Crohn’s disease (Li et al., 2016).

We have conducted a brain magnetic resonance imaging (MRI) phenome-wide association study (PheWAS) based upon the databank of different brain MRI phenotypes from the United Kingdom (UK) BioBank established previously (Elliott et al., 2018) to determine which brain MRI patterns are associated with *SLC39A8*. In addition, we performed conditional and joint association analyses on the summary statistics datasets of different MRI patterns to determine if there were independent association signals polymorphisms at the *SLC39A8* locus that were distinct from the previously reported non-synonymous polymorphism rs13107325. Given that brain structure has a heritable component (Elliott et al., 2018), it is likely that the brain structure and architecture differences may overlap with genetic effects on neurodevelopment, cognitive ability, and risks for schizophrenia. Importantly, an understanding of how polymorphisms or potentially mutations in this metal metabolism gene can affect brain structure may reveal nutritional or pharmacological approaches for optimal brain development and prevention of neuropsychiatric disorders.

## Methods

The brain MRI PheWAS was conducted using previously published data (Elliott et al., 2018) on associations between brain MRI phenotype data and genotypes using a high density array with 11,734,353 SNPs in 8,428 subjects of European ancestry in the UK Biobank. The brain MRI PheWAS was conducted through the website http://big.stats.ox.ac.uk, as made available by Elliott et al. (2018). Brain imaging and statistical analyses were described previously in detail (Elliott et al., 2018). The brain MRI PheWAS scan through 1450 different brain MRI phenotypes included 1434 structural MRI measurements and 16 task-based functional MRI assays across multiple brain regions and measures. The structural MRI datasets included 139 T1 FAST regions of interest, 22 T1 FIRST measurements, 10 T1 SIENAX measurements, 82 network-matrix analyses, 675 diffusion MRI (dMRI) analyses using either probabilistic tractography, or tract-based spatial statistics (TBSS), and 484 Freesurfer measurements of cortical thicknesses and subcortical segmentation from the Destrieux et al. (2010) or Desikan et al. (2006) atlases, and 21 susceptibility weighted T2^∗^ MRI (swMRI). We reduced the total number of MRI phenotypes by retaining only the total or bilateral measurements when left, right, and total datasets were available. This reduction removed 4 FreeSurfer, 14 swMRI, 16 T1 FAST, and 14 T1 FIRST MRI phenotypes. After this reduction, there were 1402 brain MRI phenotypes remaining. Brain MRI phenotypes at the *SLC39A8* locus were denoted as significant if the lead SNP was within 500 kilobases (kb) of the *SLC39A8* gene and had a *p*-value < 5.0 × 10^–8^. Nearby genes that also fit this criteria include *BANK1*, *MANBA*, *NFKB1*, and *UBE2D3*.

To identify the independent association signals present at each locus, we used GCTA-COJO software to perform conditional and joint association analyses (Yang et al., 2012) across Chromosome 4 as we have performed previously for Chromosome 10 (Strong et al., 2020). Summary statistic datasets for the 24 brain MRI phenotypes were downloaded from the website http://big.stats.ox.ac.uk as provided by Elliott et al. (2018). The UK10K reference panel (*n* = 3715, from Wellcome Sanger Institute) was used as the linkage disequilibrium dataset. GCTA-COJO parameters were set as *p*-value less than 1.0 × 10^–5^ for SNPs to be included in the association analyses and 10 million bases (Mb) as the analysis window.

To determine possible polymorphisms in LD in each index variant and that may be causative in altering *SLC39A8* expression or ZIP8 activity, we used SNPclip (Machiela and Chanock, 2015) set to the EUR 1000 Genomes linkage disequilibrium panel and denoted all SNPs in high LD (*r*^2^ > 0.8) with the index SNP. Missense SNP rs13107325 has been previously identified as having reduced biochemical activity and being associated with other disorders (Carrera et al., 2012; Schizophrenia Working Group of the Psychiatric Genomics Consortium, 2014; Li et al., 2016; Pickrell et al., 2016; Haller et al., 2018). Accordingly, we categorized SNPs in high LD (*r*^2^ > 0.8) with rs13107325 as being associated with rs13107325. We identified additional, independent SNPs not associated with rs13107325 and confirmed that these variants were not in LD with rs13107325 using LDpair (Machiela and Chanock, 2015) and the EUR 1000 Genomes LD panel.

Non-coding variants detected as independent association signals were examined by the PsychENCODE (Wang et al., 2018), Genotype-Tissue Expression (GTEx; GTEx Consortium, 2015), and RegulomeDB (Boyle et al., 2012) databases for possible data in gene regulation within the brain or other tissues. PsychENCODE, GTEx, and RegulomeDB were accessed at http://resource.psychencode.org, http://gtexportal.org, and http://regulomedb.org, respectively.

The statistical cutoff for significance was set at 5.0 × 10^–8^ (Fadista et al., 2016). Because of the 1402 brain MRI phenotypes in the PheWAS, we specified 3.566 × 10^–11^ as an additional, more stringent cutoff adjusted for Bonferroni correction for multiple comparisons (Denny et al., 2010). Because it is likely that the differences between brain MRI phenotypes are inter-related, rather than completely independent (Elliott et al., 2018), accounting statistically for multiple comparisons may not be necessary.

Haploblocks were drawn using LDmatrix (Machiela and Chanock, 2015) for rs13107325, associated SNPs rs13135092, rs35225200, and rs35518360, and 4 variants (rs62327945, rs201081507, rs6855246, and rs13136118) not associated with rs13107325. The LD panel used was 1000 Genomes European (EUR) set.

Rare variant data was obtained from whole exome datasets of European ancestry in the UK Biobank with brain MRI data (Cirulli et al., 2020) as we have performed previously (Strong et al., 2020). A collapsing method (Li and Leal, 2008) added all variants with a MAF less than 0.001 and included coding or splice variants predicted by SIFT or PolyPhen-2 to be not tolerated or not benign and loss of function variants including frameshift, stop gain, start lost, splice acceptor, and splice donor mutations. Data for *SLC39A8* and *BANK1* was obtained directly from the website https://ukb.research.helix.com (Cirulli et al., 2020). For *SLC39A8*, the selected brain MRI phenotypes corresponded to the 24 different brain MRI phenotypes associated with missense rs13107325 or 2 other non-coding SNPs (rs13136118 and rs201081507) that exceeded significance. The 3 brain MRI phenotypes analyzed for *BANK1* rare variants corresponded to the MRI types associated with non-coding SNPs rs13136118 and rs201081507, which were located closer to *BANK1* than *SLC39A8*. The significance of associations between the total number of coding or loss of function variants and brain MRI phenotypes for brain region and hemisphere was set at *p* < 0.05.

## Results

We found that SNPs at or near the *SLC39A8* locus were associated with 24 different brain MRI phenotypes. SNPs that are in high LD with rs13107325 (Supplementary Figure 1, *r*^2^ > 0.85) are linked to 22 brain MRI phenotypes (Table 1, *p*-value < 5.0 × 10^–8^). Out of those 22, 11 also remained significant after correction for multiple comparisons (Table 1, top 11 MRI phenotypes, *p*-value < 3.566 × 10^–11^). We detected two additional SNPs, rs6855246 and rs13136118, which are associated with 2 more MRI phenotypes (Table 2). Importantly, rs62327945, rs201081507, rs6855246, and rs13136118 remained significant (*p*-value < 5.0 × 10^–8^) after conditional and joint association testing, although only rs62327945 exceeded the more stringent threshold of 3.566 × 10^–11^. SNP rs6855246 is in moderate LD with rs13107325 (Supplementary Figure 1, *r*^2^ = 0.586), whereas rs13136118, rs201081507, and rs62327945 were not in LD with rs13107325 (Supplementary Figure 1, *r*^2^ < 0.1).

**TABLE 1:** Conditional and joint association analyses for 22 different brain MRI phenotypes associated with 22 different brain MRI phenotypes.

MRI type (and atlas)	Brain region	Hemisphere	Index SNP	LD to rs13107325	freq	*b*	se	*p*	bJ	bJ_se	pJ
T1 FAST	Putamen	Left	rs13107325	1.00	0.074	0.370	0.028	*6.76E–39*	0.347	0.029	1.60E–33
T1 FAST	Putamen	Right	rs13107325	1.00	0.074	0.367	0.028	*1.70E–39*	0.348	0.028	1.37E–34
swMRI	Pallidum	Combined	rs35225200	0.85	0.084	–0.594	0.054	*2.63E–28*	–0.546	0.054	9.83E–24
T1 FAST	Cerebellum IX	Combined	rs13107325	1.00	0.074	0.265	0.025	*5.25E–26*	0.265	0.025	7.42E–26
T1 FAST	Cerebellum X	Combined	rs13107325	1.00	0.074	0.234	0.027	*1.17E–17*	0.234	0.027	1.50E–17
dMRI TBSS ICVF	Cerebral Peduncle	Left	rs13135092	0.92	0.082	0.226	0.029	*2.24E–15*	0.226	0.029	2.71E–15
dMRI TBSS ICVF	Cerebral Peduncle	Right	rs13135092	0.92	0.082	0.212	0.028	*4.27E–14*	0.212	0.028	5.07E–14
dMRI TBSS OD	Cerebral Peduncle	Right	rs35518360	0.85	0.084	0.193	0.029	*1.48E–11*	0.193	0.029	1.54E–11
dMRI TBSS OD	Cerebral Peduncle	Left	rs35518360	0.85	0.084	0.190	0.028	*1.70E–11*	0.190	0.028	1.69E–11
swMRI	Caudate	Combined	rs13107325	1.00	0.074	–0.368	0.055	*2.19E–11*	–0.368	0.055	2.29E–11
FreeSurfer (Destrieux)	Occipital Pole (thickness)	Right	rs13107325	1.00	0.074	–0.190	0.029	*3.24E–11*	–0.190	0.029	3.31E–11
T1 FAST	Cerebellum VIIIa	Combined	rs35225200	0.85	0.084	0.151	0.024	2.14E–10	0.151	0.024	2.22E–10
FreeSurfer (Destrieux)	Cuneus Gyrus (thickness)	Right	rs13107325	1.00	0.074	–0.186	0.029	2.00E–10	–0.186	0.029	2.20E–10
T1 FAST	Cerebellum VIIIb	Combined	rs13135092	0.92	0.082	0.148	0.024	8.32E–10	0.148	0.024	9.44E–10
dMRI TBSS ICVF	Anterior Limb of Internal Capsule	Left	rs13107325	1.00	0.074	0.174	0.029	1.91E–09	0.176	0.029	1.36E–09
FreeSurfer (Desikan)	Lateraloccipital lobe (thickness)	Right	rs13107325	1.00	0.074	–0.167	0.029	6.46E–09	–0.167	0.029	6.34E–09
dMRI TBSS ICVF	Anterior Limb of Internal Capsule	Right	rs13107325	1.00	0.074	0.160	0.029	2.14E–08	0.161	0.029	1.58E–08
FreeSurfer (Destrieux)	Sup Occipital Gyrus (thickness)	Right	rs13107325	1.00	0.074	–0.162	0.029	2.75E–08	–0.162	0.029	2.85E–08
FreeSurfer (Destrieux)	Occipital Pole (thickness)	Left	rs35518360	0.85	0.084	–0.154	0.028	3.39E–08	–0.154	0.028	3.60E–08
T1 FAST	Cerebellum VI	Left	rs13135092	0.92	0.082	0.124	0.023	3.63E–08	0.124	0.023	3.86E–08
FreeSurfer (Desikan)	White Matter (surface area)	Right	rs13107325	1.00	0.074	0.079	0.014	3.63E–08	0.079	0.014	4.09E–08
FreeSurfer (Desikan)	Lateraloccipital lobe (thickness)	Left	rs13135092	0.92	0.082	–0.149	0.027	4.27E–08	–0.149	0.027	4.27E–08

*Index single nucleotide polymorphisms (SNP) are indicated by rsID. LD of index SNP to rs13107325 listed. SNPs meeting *p*-value threshold for significance (less than 5.0 × 10^–8^) prior to association analyses are listed. Italics indicate *p*-values less than 3.566 × 10^–11^ (threshold after Bonferroni correction). Minor allele frequency (freq), unadjusted beta (*b*), standard error (se), and *p*-values (*p*) from the original GWAS summary statistics dataset was provided by Elliott et al. (2018). Corresponding adjusted beta (bJ), standard error (bJ_se), and *p*-values (pJ) following conditional and joint (COJO) association analyses by GCTA-COJO are provided.*

**TABLE 2:** Conditional and joint association analyses for 5 different brain MRI phenotypes associated with SNPs distinct from (and in linkage equilibrium with) rs13107325.

MRI type	Brain region	Hemisphere	Index SNP	freq	b	se	*p*	bJ	bJ_se	pJ
swMRI	Pallidum	Combined	rs62327945	0.093	0.465	0.050	9.12E–21	0.410	0.050	2.88E–16
T1 FAST	Putamen	Left	rs201081507^#^	0.053	0.264	0.036	1.45E–13	0.202	0.036	2.22E–08
T1 FAST	Putamen	Right	rs201081507^#^	0.053	0.259	0.035	1.78E–13	0.202	0.036	1.3E–08
T1 FAST*	Brain Stem*	Combined (Whole)	rs6855246	0.078	0.144	0.026	1.51E–08	0.144	0.026	1.63E–08
dMRI TBSS OD*	Anterior Limb of Internal Capsule*	Left	rs13136118^#^	0.223	0.106	0.019	1.55E–08	0.108	0.019	1.15E–08

Index single nucleotide polymorphisms (SNP) are indicated by rsID. SNPs meeting *p*-value threshold for significance (less than 5.0 × 10^–8^) prior to association analyses are listed. Minor allele frequency (freq), unadjusted beta (b), standard error (se), and *p*-values (*p*) from the original GWAS summary statistics dataset was provided by Elliott et al. (2018). Corresponding adjusted beta (bJ), standard error (bJ_se), and *p*-values (pJ) following conditional and joint (COJO) association analyses by GCTA-COJO are provided. Asterisk (*) indicates a brain MRI phenotype not in Table 1 and not associated with rs13107325. Pound sign (#) indicates a variant that is closer to *BANK1* than *SLC39A8*.

The rs13107325 variant was associated with volumetric brain region differences found by T1-weighted FAST imaging, which detects gray brain matter (Elliott et al., 2018; Jiang et al., 2018). Differences in gray matter volume associated with rs13107325 were observed in the putamen and lobules VI, VIIIa, VIIIb, IX, and X of the cerebellum (Table 1). Lobules VI and VIIIa through X occupy the posterior and flocculonodular lobes of the cerebellum. In addition to rs13107325, rs201081507 was also associated with differences in gray matter volume of the putamen. SNP rs6855246 was associated with more brain stem gray matter (Table 2), whereas the missense polymorphism rs13107325 failed to reach significance for this brain MRI phenotype.

In addition to differences in gray matter throughout the brain, we detected variants associated with regional differences in brain metal content, connectivity, cortical thickness, and white matter surface area. rs13107325 was associated with differences in swMRI intensity, which is greatly affected by brain metal content (Liu et al., 2015; Elliott et al., 2018), in the caudate and the pallidum (Table 1). We detected an additional, independent variant, rs62327945, that is associated with pallidum swMRI (Table 2). rs13107325 is associated with differences in connectivity, as measured by TBSS. Greater orientation dispersion (OD) and intracellular volume fraction (ICVF), which are measures of neurite orientation and neurite density, respectively, were found in the cerebral peduncles (OD and ICVF, Table 1). Increased neurite density was associated with rs13107325 in the anterior limb of the internal capsule (ICVF only, Table 1). rs13136118 was also associated with greater orientation dispersion in the left anterior limb of the internal capsule (Table 2). rs13107325 was associated with reduced cortical thickness in the occipital region and increased white matter surface area in the right hemisphere, as detected by Freesurfer analyses (Desikan et al., 2006). Differences in cortical thicknesses were measured in occipital pole, cuneus gyrus, superior occipital gyrus, and lateral occipital lobe, which are within the occipital region of the brain. The thickness of the cortical layer across these different regions in the occipital and cuneus areas was thinner in subjects with the rs13107325 SNP.

Non-coding SNPs rs62327945, rs201081507, rs6855246, and rs13136118 were examined for possible roles in gene regulation by querying the PsychENCODE (Wang et al., 2018), GTEx (GTEx Consortium, 2015), and RegulomeDB (Boyle et al., 2012) databases. Variant rs13136118 was an expression quantitative trait locus (eQTL) for *SLC39A8*, as determined through PsychENCODE (Wang et al., 2018). However, the GTEx database showed that rs13136118 was an eQTL in thyroid, spleen, lung, and whole blood, but not for brain. SNP rs6855246 is listed as an eQTL for cultured fibroblasts in GTEx. The RegulomeDB indicated that SNP rs62327945 was located at a binding site for transcription factors FOS and STAT3, as determined by chromatin immunoprecipitation assay. In addition, SNP rs201081507 was located within a motif that matches the response element for metal-responsive MTF-1, although there was no experimental data provided to support whether this site was biologically active.

Subjects with rare, presumably deleterious *SLC39A8* variants showed differences in many brain MRI phenotypes affected by rs13107325. Subjects with rare ZIP8 variants had differences in right putamen and cerebellar gray matter volumes and left pallidum swMRI intensity (Table 3 and Supplementary Dataset 1). In these brain regions, the mutations appeared to have a greater impact, as quantified by beta, than rs13107325, which may be due to the predicted severity and loss of function for these mutations. One limitation is that only 11–14 ZIP8 coding and loss-of-function mutations were present in the study pool of 9286–10415 subjects, so that there were insufficient individuals with solely loss-of-function mutations to be analyzed as a separate group. The underrepresentation of deleterious *SLC39A8* mutations had also been observed in 60,706 subjects from the Exome Aggregation Consortium (Lek et al., 2016), which was consistent with pathogenicity of ZIP8 mutations leading to a genetic disorder with a recessive inheritance pattern (Boycott et al., 2015; Park et al., 2015).

**TABLE 3:** *SLC39A8* rare variants and brain MRI phenotypes.

MRI type	Brain region and hemisphere		Freq variants	*N* variants	*N* subjects	Beta	SE	*p*-val (inf)	*p*-val (non-inf)
T1 FAST	Putamen	Left	0.000672	14	10415	0.504	0.262	0.054	0.069
T1 FAST	Putamen	Right	0.000672	14	10415	0.710	0.260	**0.0063**	**0.0076**
swMRI	Pallidum	Left	0.00068	13	9565	–0.464	0.270	0.086	**0.045**
swMRI	Pallidum	Right	0.00068	13	9565	–0.264	0.276	0.34	0.31
T1 FAST	Cerebellum IX	Left	0.000672	14	10415	0.450	0.262	0.086	0.076
T1 FAST	Cerebellum IX	Right	0.000672	14	10415	0.541	0.262	**0.039**	**0.038**
T1 FAST	Cerebellum X	Left	0.000672	14	10415	0.414	0.244	0.089	0.089
T1 FAST	Cerebellum X	Right	0.000672	14	10415	0.141	0.244	0.56	0.55
dMRI TBSS ICVF	Cerebral Peduncle	Left	0.000592	11	9286	0.317	0.300	0.29	0.27
dMRI TBSS ICVF	Cerebral Peduncle	Right	0.000592	11	9286	0.348	0.295	0.24	0.22
dMRI TBSS OD	Cerebral Peduncle	Left	0.000592	11	9286	0.124	0.291	0.67	0.67
dMRI TBSS OD	Cerebral Peduncle	Right	0.000592	11	9286	0.126	0.293	0.67	0.67
swMRI	Caudate	Left	0.00068	13	9565	–0.275	0.269	0.31	0.3
swMRI	Caudate	Right	0.00068	13	9565	–0.105	0.272	0.7	0.62
T1 FAST	Cerebellum VIIIa	Left	0.000672	14	10415	0.593	0.260	**0.023**	**0.024**
T1 FAST	Cerebellum VIIIa	Right	0.000672	14	10415	0.568	0.261	**0.029**	**0.029**
T1 FAST	Cerebellum VIIIb	Left	0.000672	14	10415	0.584	0.260	**0.025**	**0.023**
T1 FAST	Cerebellum VIIIb	Right	0.000672	14	10415	0.891	0.261	**0.00063**	**0.00059**
dMRI TBSS ICVF	Anterior Limb of Internal Capsule	Left	0.000592	11	9286	–0.037	0.280	0.89	0.91
dMRI TBSS ICVF	Anterior Limb of Internal Capsule	Right	0.000592	11	9286	–0.162	0.278	0.56	0.53
T1 FAST	Cerebellum VI	Left	0.000672	14	10415	0.326	0.251	0.19	0.19

*MRI phenotypes for rare variants of *SLC39A8* are ordered by significance for rs13107325. MRI phenotypes include susceptibility-weighted MRI (swMRI; measure of mineral content), T1 FAST (gray matter volume), and dMRI TBSS ICVF and OD [diffusion MRI tract-based spatial statistics, intracellular volume fraction (axonal density) and orientation dispersion (neurite dispersion), respectively]. Beta, standard error (SE), and *p*-values were obtained directly from data analyzed by Cirulli et al. (2020). Beta indicates variances associated with combined coding and loss-of-function variants in *SLC39A8* exomes under a collapsing model. BOLT-LMM *p*-values were obtained from infinitesimal (inf) and non-infinitesimal (non-inf) models during mixed model association testing as performed by Cirulli et al. (2020). Boldface indicates p-values less than 0.05.*

We found non-coding polymorphisms located physically closer to *BANK1* than *SLC39A8* that were also linked to differences in brain structure. rs201081507 is associated with gray matter in the putamen and lies 31 kb from the *BANK1* transcription start site and more than 450 kb from the *SLC39A8* gene. Variant rs13136118 is located within an intron for *BANK1*. We hypothesized that if *BANK1* does not affect brain structures associated with rs201081507 and rs13136118, respectively, then rare, deleterious mutations in *BANK1* would not alter these brain MRI phenotypes. Rare heterozygous variants in *BANK1* predicted to have reduced or complete loss of function are not associated with gray matter volumes in the putamen or neurite dispersion in the anterior limb of interior capsule (Table 4, *p* > 0.05), although no homozygous *BANK1* mutations were present in the sequenced individuals.

**TABLE 4:** *BANK1* rare variants and select brain MRI phenotypes.

Coding model								
MRI type	Brain region and hemisphere		Freq variants	*N* variants	Beta	SE	*p*-val (inf)	*p*-val (non-inf)
T1 FAST	Putamen	Left	0.003889	81	0.082848	0.109086	0.45	0.43
T1 FAST	Putamen	Right	0.003889	81	0.094178	0.108313	0.38	0.37
dMRI TBSS OD	Anterior Limb of Interior Capsule	Left	0.003715	69	–0.00889	0.119939	0.94	0.93

**Loss of function model**								

**MRI type**	**Brain region and hemisphere**		**Freq variants**	***N* variants**	**Beta**	**SE**	***p*-val (inf)**	***p*-val (non-inf)**

T1 FAST	Putamen	Left	0.0012	25	0.19048	0.195826	0.33	0.34
T1 FAST	Putamen	Right	0.0012	25	0.295575	0.194437	0.13	0.13
dMRI TBSS OD	Anterior Limb of Interior Capsule	Left	0.001185	22	0.115032	0.211874	0.59	0.6

*MRI phenotypes for rare variants of *BANK1* are matched to rs6855246 and rs13136118, which are not in LD with rs13107325. MRI phenotypes include T1 FAST (gray matter volume) and dMRI TBSS OD [diffusion MRI tract-based spatial statistics, orientation dispersion (neurite dispersion)]. Beta, standard error (SE), and *p*-values were obtained directly from data analyzed by Cirulli et al. (2020). Beta indicates variances associated with combined coding and loss-of-function variants, or restricted to only loss-of-function variants, in *BANK1* exomes under a collapsing model. BOLT-LMM *p*-values were obtained from infinitesimal (inf) and non-infinitesimal (non-inf) models during mixed model association testing as performed by Cirulli et al. (2020). No *BANK1* rare variants achieve significance (*p* > 0.05 for all MRI phenotypes).*

## Discussion

Differences in gray matter volume associated with rs13107325 were observed in the putamen and multiple lobules of the cerebellum. Previous research reported that rs13107325 is associated with greater amounts of gray matter in the putamen across the lifespan (Luo et al., 2019). Patients with schizophrenia have larger gray matter volumes in the putamen (Antonova et al., 2005; Cui et al., 2011; Knochel et al., 2016). In our study, the link between rs13107325 and putamen gray matter appears to be stronger than for cerebellar gray matter, as measured by larger overall effect size (beta) and lower inter-person variability (standard error of beta and *p*-value). A comparison between monozygotic and dizygotic twins shows that cerebellar volume and cerebral white matter are heritable and positively correlated (Brouwer et al., 2014a). More investigation is needed to determine if SNPs in *SLC39A8* impact both putamen and cerebellar development through a common pathway or if the effect of *SLC39A8* on these brain regions occur independently of each other.

There are detectable differences in the brain stem of patients with schizophrenia (Hayashida et al., 1986; Lindstrom et al., 1987). Auditory brain stem response abnormalities have been found in schizophrenic patients with auditory hallucinations, in comparison to patients without hallucinations (Lindstrom et al., 1987). Schizophrenic patients with marked deterioration are more likely to have auditory brain stem responses lacking portions of the auditory brain stem response waveforms (Hayashida et al., 1986). Another brain MRI GWAS with more participants (27,034) from the UK Biobank was able to detect an association between rs13107325 and medulla oblongata volume (Elvsashagen et al., 2020). More studies are needed to determine whether other SNPs, particularly non-coding variants, may affect brain subregions that are distinct from missense SNP rs13107325.

Because *SLC39A8* polymorphisms are associated with differences in swMRI intensity in the basal ganglia, and swMRI intensity is greatly affected by mineral content, it is presumed that subjects with these SNPs are likely to have altered brain metal homeostasis. ZIP8 has been shown to transport manganese, zinc, and cadmium in addition to iron (Dalton et al., 2005; Wang et al., 2012). In patients with *SLC39A8* mutations, cerebral atrophy is present and T2-weighted MRI differences are observable in basal ganglia (Riley et al., 2017). However, it is unclear whether the differences in swMRI intensity associated with rs13107325 are due to altered manganese transport or the impaired homeostasis of other minerals such as iron. The iron buildup by the basal ganglia is particularly noticeable in neurodegeneration with brain iron accumulation (Schipper, 2012). swMRI can detect brain microbleeds and iron deposits (Liu et al., 2015; Elliott et al., 2018). In contrast, brain iron measurement via R2^∗^ MRI relaxometry in children shows that increased brain iron is associated with a faster processing speed and higher overall intelligence (Hect et al., 2018). If the differences in swMRI intensity associated with rs13107325 are due to altered iron transport, it is possible that high brain iron may promote brain development early in life but increase susceptibility to neurodegeneration or brain aging in later adulthood. Polymorphisms and rare inactivating mutations in zinc transporter ZIP12 are associated with swMRI differences in the basal ganglia (Strong et al., 2020), which suggests that altered intracellular zinc dynamics may also impact brain swMRI intensity. More studies are needed to determine what metals are responsible for the swMRI differences due to ZIP8 polymorphisms.

*SLC39A8* polymorphisms are associated with measures of altered neural connectivity. TBSS of dMRI is used as a measure of structural connectivity (Elliott et al., 2018; Alexander et al., 2019). ICVF detects neurite density, and OD reflects the spreading or fanning of neurite structures (Cox et al., 2016). Smaller internal capsule size is found in schizophrenic patients with poor outcomes in comparison to subjects with good outcomes (Brickman et al., 2006). White matter integrity measured by dMRI TBSS is reduced in treatment-resistant schizophrenia compared to healthy controls and treatment-responsive patients (Ochi et al., 2020). Connectivity as measured by dMRI TBSS is linked to faster information processing speed in older adults, although these associations did not apply to cognitive ability measured in childhood (Kuznetsova et al., 2016).

There were also differences in cortical thickness in the occipital region and altered white matter surface area associated with polymorphisms in *SLC39A8*. Differences in cortical thicknesses were measured in the occipital pole, cuneus gyrus, superior occipital gyrus, and lateral occipital lobe, which are all within the occipital region of the brain. Reductions in cortical thickness have been reported in patients with schizophrenia (Wannan et al., 2019). In another study, reductions in cortical thickness were also observed in patients with schizophrenia (van Erp et al., 2018). In both studies, the thinness was most pronounced in the frontal and temporal cortical layers of patients with schizophrenia. Furthermore, healthy adult subjects from the UK Biobank with higher polygenic risk scores for schizophrenia are more likely to have thinner cortices at the frontotemporal lobes (Alnaes et al., 2019). Cortical thickness is associated with intelligence and mediated by genetics (Schmitt et al., 2019), although these genetic associations are present starting at puberty and not in early childhood (Brouwer et al., 2014b). White matter surface area was larger in subjects with the rs13107325 SNP. These measurements in occipital cortical thickness and white matter surface area passed the 5.0 × 10^–8^ *p*-value cutoff but failed or barely met the more stringent *p*-value threshold of 3.566 × 10^–11^. It is possible that there is heterogeneity in occipital cortical thickness among those with the rs13107325 SNP. Elliott et al. (2018) have stated that adjustments for multiple comparisons may not be necessary given that the brain MRI phenotypes are likely interlinked and not independent. Others have mentioned that a Bonferroni correction may be too conservative and reduce sensitivity (Pendergrass et al., 2011). Future studies can determine if these associations in occipital cortical thickness and white matter surface area can be replicated in other sample populations.

There is little published information about other polymorphisms in ZIP8, including the non-coding variants that were detected as novel associations in this study. Although the population in this study is restricted to subjects of European descent from the UK Biobank, it is possible that *SLC39A8* is a risk factor in other populations as well. For example, SNP rs10014145 in *SLC39A8* is associated with schizophrenia in Han and Uygur Chinese (Jian et al., 2019). In addition, a different coding SNP rs11097773 in *SLC39A8* in Han Chinese has been associated with scoliosis (Xu et al., 2020), although that study did not find an association between rs13107325 and scoliosis. The rs13107325 is relatively under-represented in populations outside of European origins (Carrera et al., 2012). The novel variants detected in this study, rs62327945 and rs201081507, are equally prevalent across different populations, whereas rs6855246 is most prevalent in African populations and rs13136118 is found more often in European and East Asian populations (Genomes Project Consortium, Abecasis et al., 2012). In summary, future investigation of genetic variation in *SLC39A8* may reveal how these novel variants may affect gene regulation, metal metabolism, and possibly disease risk linked to ZIP8 function.

Out of the SLC39 gene family, only SLC39A8 is associated with a wide variation of brain MRI phenotypes. Of the 14 ZIP members, only ZIP8, ZIP12, and ZIP14 have brain MRI phenotypes that meet the significance threshold set for this PheWAS. ZIP12 is linked to swMRI intensity in the caudate, putamen, and pallidum and to gray matter putamen (Strong et al., 2020), which is similar to the brain MRI phenotypes tied to ZIP8. ZIP14 is associated with differences in gray matter (T1 FAST) in the right ventral striatum and cortical thickness (Freesurfer) of the left orbital lateral area. Mutations in ZIP14 cause childhood-onset parkinsonism-dystonia, elevated blood Mn levels, and T1-hyperintensity in the globus pallidus and striatum (Tuschl et al., 2016). The impact of ZIP8 and ZIP14 on brain MRI phenotypes may be related to the added permeability of these specific ZIP transporters to manganese and iron (He et al., 2006; Liuzzi et al., 2006; Wang et al., 2012; Tuschl et al., 2016), whereas most ZIP transporters are specific for zinc (Eide, 2004). The impact of ZIP12 on brain structure and MRI phenotypes may be traced to the high expression of ZIP12 in the nervous system (Chowanadisai et al., 2013; Chowanadisai, 2014). The wide extent of brain differences due to ZIP8 polymorphisms is likely due to reduced manganese-dependent β-1,4-galactosyltransferase activity and widespread hypoglycosylation in the proteome (Boycott et al., 2015; Park et al., 2015; Lin et al., 2017).

One limitation is that the conclusions from this study are based solely on the UK Biobank data. Additional studies in which replication cohorts are examined will provide a future direction for confirming the observed associations between *SLC39A8* polymorphisms and brain structure and MRI phenotypes in this study. In a voxelwise GWAS study performed in 1721 healthy adolescent 14 year olds, *SLC39A8* polymorphism rs13107325 was found to be associated with a larger gray matter volume of the putamen (Luo et al., 2019). The authors also found similar findings in replication cohorts from the 272 in the Lieber Institute for Brain Development sample, 515 healthy elderly in the Three-City Study (3C20), and 6932 older adults (62 years old average) in the UK Biobank cohort, which likely overlaps with our findings here for putamen gray matter volumes measured by T1 FAST MRI. The findings between *SLC39A8* and putamen gray matter are strengthened by the use of replication cohorts, including data from the UK Biobank.

In summary, the data from this MRI-based PheWAS provides information about brain differences associated with brain function and identifies other polymorphisms in the same gene which may also result in structural brain differences. This data provides leads about how genetic variants in a metal metabolism gene may affect brain structure, associated cognition ability, and risks for psychiatric disorders. It is also possible that manipulation of dietary manganese intake may be an intervention strategy by which brain function can be promoted and protected across the lifespan in response to polymorphism in the *SLC39A8* gene.

## Data Availability Statement

The original contributions presented in the study are included in the article/Supplementary Material, further inquiries can be directed to the corresponding author/s.

## Ethics Statement

Ethics approval for the use of human data in this study was waived by the Institutional Review Board at Oklahoma State University. All data used in the study are from existing databases and published sources, which are cited. Ethics approval for the use of animal data in this study is not applicable.

## Author Contributions

EH performed conditional and joint association analyses and database summary preparation. EC and DD performed dataset preparation and haploblock design. EH, DD, MM, DL, and WC wrote the manuscript. DL assisted with the design of the study. WC supervised and designed the project. All authors read and approved the final manuscript.

## Conflict of Interest

The authors declare that the research was conducted in the absence of any commercial or financial relationships that could be construed as a potential conflict of interest.

## Abbreviations

dMRI: diffusion magnetic resonance imaging
eQTL: expression quantitative trait locus
EUR: European
GWAS: genome-wide association study
GTEx: Genotype-Tissue Expression
ICVF: intracellular volume fraction
kb: kilobases (thousand bases)
LD: linkage disequilibrium
Mb: megabases (million bases)
MRI: magnetic resonance imaging
MTF-1: metal regulatory transcription factor-1
OD: orientation dispersion
PheWAS: phenome-wide association study
SNP: single nucleotide polymorphism
STAT-3: signal transducer and activator of transcription 3
swMRI: susceptibility-weighted magnetic resonance imaging
TBSS: tract-based spatial statistics
UK: United Kingdom.
