# Supplementary material for: Brain Magnetic Resonance Imaging Phenome-Wide Association Study With Metal Transporter Gene SLC39A8
Source: Front Genet. 2021 Mar 15;12:647946. doi: 10.3389/fgene.2021.647946 (PMC8005600; doi:10.3389/fgene.2021.647946)
Supplement: Supplementary file 1 [file Image_1.PDF]

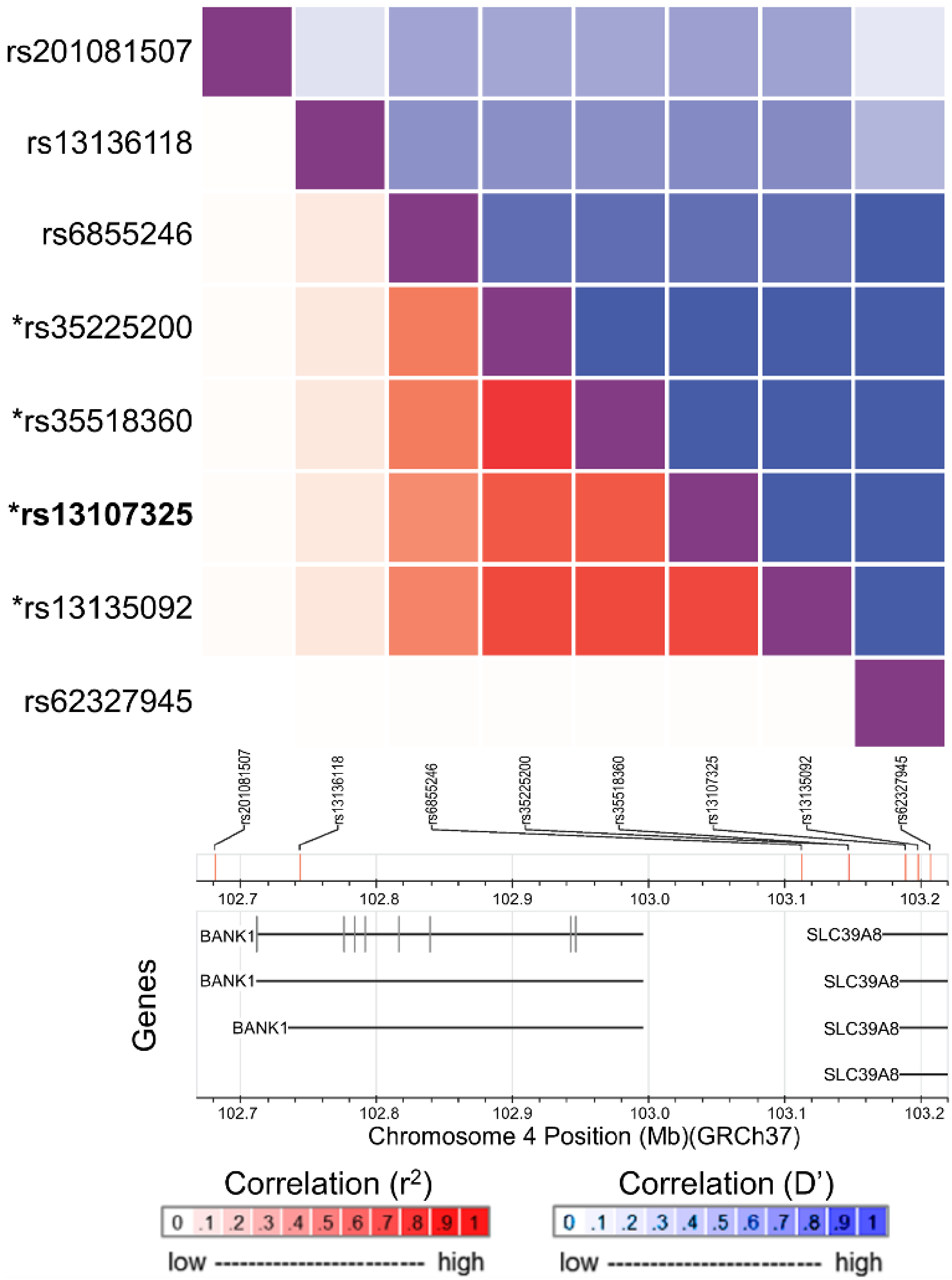

Supplementary Figure 1. Linkage disequilibrium (LD) between *SLC39A8* variant rs13107325 and additional, independent variants associated with brain MRI phenotypes, as depicted as haploblocks by LDmatrix software. Variant rs13107325 (asterisk, boldface) is in high LD with rs13135092, rs35225200, and rs35518360 (asterisks). SNPs rs62327945, rs201081507, rs6855246, and rs13136118 are in relatively low LD with rs13107325. SNPs rs201081507 and rs13136118 are located near the *BANK1* gene. The 1000 Genomes European (EUR) dataset was used as the LD reference panel. Dark red and blue blocks indicate variants in high LD (r^2 depicted in red; D' depicted in blue). White or light pink or faint blue blocks indicate variants not in LD with each other. Variants and genes *SLC39A8* and *BANK1* were mapped to coordinates on chromosome 4 (million bases, Mb) relative to the GRChr37 human genome.
